# The healthcare burden of pulmonary alveolar proteinosis (PAP)

**DOI:** 10.1186/s13023-024-03478-2

**Published:** 2025-02-14

**Authors:** Elinor Lee, Ali Ataya, Cormac McCarthy, Erica Godart, John Cosenza, Alysse King, Brian Robinson, Tisha Wang

**Affiliations:** 1 UCLA Department of Medicine, Division of Pulmonary and Critical Care Medicine, University of California Los Angeles, 650 Charles E. Young Drive South, 43-229 CHS, Los Angeles, CA 90095-1690 USA; 2 Division of Pulmonary and Critical Care Medicine, University of Florida, Gainesville, FL USA; 3 School of Medicine, University College Dublin, Dublin, Ireland; 4 IPM.Ai, a Real Chemistry Company, New York, NY USA; 5 Savara Inc, Langhorne, PA USA

**Keywords:** Burden of illness, Charlson comorbidity index (CCI), Healthcare resource utilization (HCRU), Anti-GM-CSF antibody, Whole-lung lavage

## Abstract

**Introduction:**

Pulmonary alveolar proteinosis (PAP) is a rare lung syndrome characterized by the accumulation of surfactant in the alveoli. Using a longitudinal claims database, we compared measures of clinical and economic burden between a sample of diagnosed PAP patients and non-PAP matched controls.

**Methods:**

PAP patients were identified leveraging IPM.ai’s longitudinal U.S. claims database spanning January 1, 2009, through May 1, 2022. PAP patients were selected based on the presence of ICD-10: J84.01 or ICD-9: 516.0 in their claims history and were indexed for observation. An age, gender, and geographically matched control cohort was created (ratio of 1:4) for comparison. A third cohort, consisting of likely undiagnosed PAP patients, was identified using a machine learning model. The PAP and control cohorts were tracked longitudinally, depending on individual index dates, from January 1, 2018, through May 1, 2023. Inclusion criteria required evidence of continual claims activity 12 months prior to and after the index date, which reduced the total number of diagnosed PAP and control patients in the analysis. Demographics, comorbidities, procedures, medication use, annual healthcare resource utilization (HCRU), and costs were calculated for eligible PAP and control patients and were compared 12 months prior to, and 12 months after each patient’s index date.

**Results:**

After inclusion criteria were applied, 2312 confirmed PAP patients and 9247 matched controls were included in the analysis. Compared with matched controls, PAP patients had significantly higher rates of diagnosed conditions at baseline as defined by the Charlson Comorbidity Index (CCI). During the follow-up period, PAP patients had higher rates of diagnosed conditions, procedures, medication use, and cost-of-care compared with controls. PAP patients also had higher rates of emergency room visits (35% vs. 14%; *P* < 0.001), outpatient visits (87% vs. 56%; *P* < 0.001), inpatient visits (20% vs. 5%; *P* < 0.001) and had longer lengths of stay for inpatient hospitalizations (2.8 days vs. 0.56 days; *P* < 0.001), respectively.

**Conclusion:**

This study represents the largest dataset of PAP patients and matched controls to be analyzed to date. Findings indicate that PAP patients have higher rates of diagnosed conditions, procedures, medication use, HCRU, and costs compared with non-PAP patients.

**Supplementary Information:**

The online version contains supplementary material available at 10.1186/s13023-024-03478-2.

## Introduction

Pulmonary alveolar proteinosis (PAP) is a rare lung syndrome that is characterized by the accumulation of surfactant in the alveolar space resulting in impaired gas exchange, pulmonary symptoms [1, 2], and increased susceptibility to secondary infections from common and opportunistic pathogens [3–5]. PAP is classified into primary, secondary, and congenital forms [3]. In primary PAP, there is a disruption in granulocyte–macrophage colony-stimulating factor (GM-CSF) signaling, which leads to surfactant accumulation, due to either elevated GM-CSF autoantibody levels (autoimmune) [6] or genetic mutations involving the GM-CSF receptor (hereditary) [7]. Autoimmune PAP is the most common type of PAP, accounting for approximately 90% of cases. Secondary PAP is associated with systemic disorders, such as hematological malignancies (most commonly myelodysplastic syndrome), immune deficiency syndromes, or toxic inhalational exposures that secondarily affect the number and/or function of alveolar macrophages [3]. Congenital PAP is caused by mutations in genes essential for surfactant production including surfactant-associated protein B (*SFTPB*), surfactant-associated protein C (*SFTPC*), and ATP binding cassette protein 3 (*ABCA3*) [3, 8].

Despite our understanding of PAP pathophysiology, the path to a definitive diagnosis and treatment can be challenging due to nonspecific clinical symptoms and findings, limited access to testing, and the lack of approved therapies [3, 9, 10]. A study using data from the U.S. National PAP Registry has shown a median delay of 1.5 years in accurately diagnosing PAP after symptom onset [11]. This same study showed that the diagnostic workup frequently involves multiple tests and procedures, including pulmonary function tests, arterial blood gas analysis, chest radiographs, CT scans, and examination of BAL cytology and/or lung histopathology. In addition, results from the U.S. National PAP Registry revealed that many patients with PAP undergo transbronchial biopsy, surgical lung biopsy, or both during their diagnostic journey [11].

Whole lung lavage (WLL), the current standard of care for PAP, is an invasive procedure that physically removes excess surfactant and is neither standardized nor widely available [2]. Although it alleviates symptoms, the benefits of WLL are short-lived, and the majority of patients often need multiple WLLs to manage the disease. Treatment of autoimmune PAP with recombinant GM-CSF augmentation was first described in 1996 and has been used off-label [12–14]. Other treatment approaches include plasmapheresis, rituximab, statins, and lung transplantation [15–18].

Taken together, these challenges result in significant healthcare burden to PAP patients, their families, and the healthcare system. Increased understanding and awareness of PAP, increased patient screening through early appropriate testing, approved treatments that address the underlying disease pathology, and the development of clinical practice guidelines, are all important steps toward alleviating some of this healthcare burden. Previous research conducted on a limited subset of the U.S. population demonstrated higher comorbidities, increased healthcare utilization, and elevated costs in PAP patients compared with controls [9]. Here, we set out to validate and expand upon these findings within a larger U.S. cohort between a subset of diagnosed PAP patients and matched non-PAP control patients. Additionally, we employed machine learning (ML) technology to identify additional patients in the healthcare system that exhibit indicators of PAP, which may support the notion of an underdiagnosis of PAP as suggested in other studies.

## Methods

### Data source

This study used IPM.ai’s integrated longitudinal claims database that spans from January 1, 2009, through present day covering approximately 300 million active patient lives across the United States. IPM.ai’s dataset is a de-identified, comprehensive, open-sourced claims database that includes medical, hospital, prescription, and specialty pharmacy claims captured across the healthcare system.

### Study design and populations

A retrospective cohort analysis was performed using claims data captured during a study period from January 1, 2018, through May 1, 2023. Patients with PAP were included in the study if they had:

1.  ≥ 1 claim with a diagnosis code for PAP (ICD-9-CM code: 516.0 or ICD-10-CM code: J84.01) during an index period between January 1, 2019, and May 1, 2022, and
2. No claims for other rare respiratory diseases (See Supplemental Data: Table E1 for diagnosis codes) occurring after the last PAP diagnosis code during the study period.

The date of the earliest claim associated with a PAP diagnosis code was assigned as the index date for PAP patients with a PAP diagnosis code during the index period. If PAP patients did not have a claim with a PAP diagnosis code in the index period, they were assigned an index date of January 1, 2019, the first day of the index period.

A non-PAP control cohort was created using a 1:4 (case:controls) ratio for comparison. To identify the controls, each PAP patient was matched to 4 patients within IPM.ai’s claims database based on age, gender, and geographic location. Patients in the control cohort were assigned the same index date as their matched PAP patient.

Matching was used to reduce variability and systematic differences between the groups. Age and gender were utilized because these are strong confounders of disease. As such, these matched controls provide a comparison to similar demographic populations in the same claims data. Given their visibility in the claims data, these control patients were also using the healthcare system and thus unlikely to be perfectly healthy. Furthermore, the 1:4 (case:controls) was used to increase the precision of statistical analyses by including more than one control for each patient.

**Fig. 1:**
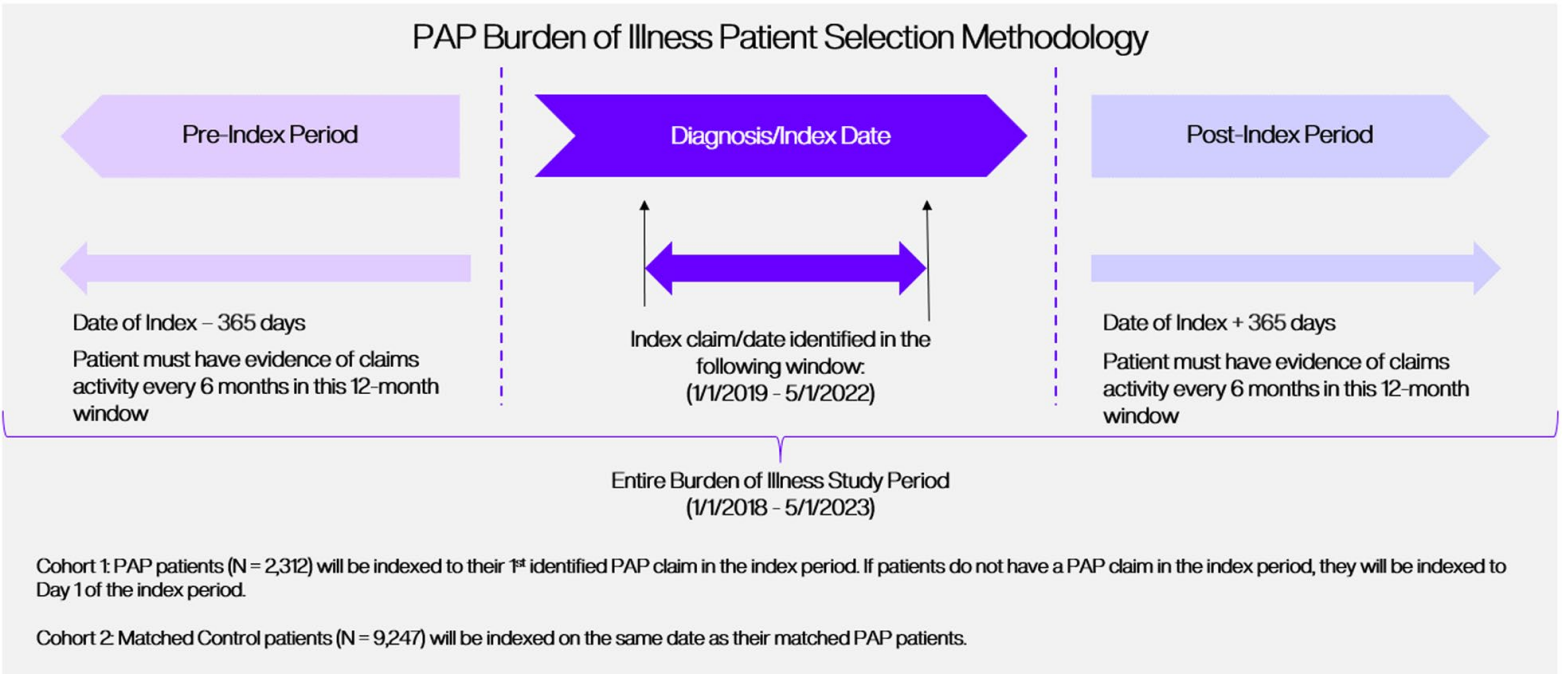
Patient inclusion criteria

To be included in the analysis, PAP and control patients must have had evidence of continual claims activity (≥ 1 claim in two 6-month windows) in both the 12 months prior to (baseline period) and after (follow-up period) the index date (Fig. 1). These additional criteria determined which patients were included in the analysis. Any diagnosed PAP or control patients who did not meet these criteria were excluded from the analysis.

Additionally, IPM.ai’s machine learning (ML) technology [19, 20] was applied to identify likely undiagnosed PAP patients. The ML algorithm was developed based on a set of “training” data from a cohort of diagnosed PAP patients. The training data were based on diagnosed patients who had the following:

1.  ≥ 2 claims with a diagnosis code for PAP (ICD-9-CM code: 516.0 or ICD-10-CM code: J84.01) in their entire claims history.
2. No claims for other rare respiratory diseases (See Supplemental Data: Table E1 for diagnosis codes) occurring after the last PAP diagnosis code captured in their claims history.

The ML algorithm analyzed the entire claims history of the training cohort and identified patterns across their diagnosis, medical, and treatment claims unique to PAP patients. Based on these identified patterns, the ML algorithm scored the remaining patients in IPM.ai’s ~ 300 million patient universe. Our ML cohort included patients who received the highest scores, indicating a resemblance to diagnosed PAP patients. Moreover, evidence of procedures commonly seen among the training cohort (bronchoscopy, bronchoalveolar lavage [BAL], or total lung lavage) was required to be present in their claims history. These procedures were leveraged as filtering criteria to limit the rate of false-positive predictions, and thus, ensured that patients predicted from the ML algorithm further resembled diagnosed PAP patients. A variety of metrics were used to objectively evaluate the ML model (area under the curve [AUC], precision, recall, and performance vs a baseline model), which allowed us to identify and optimize the most appropriate predictive ML model. In comparing the success of the ML model in finding likely PAP patients from the “training” data, the ML model produced an AUC of 0.89, indicating very strong predictive behavior to effectively identify potentially undiagnosed PAP patients. Nevertheless, given potential limitations of the algorithm, the ML cohort was not included in the main analysis of this study.

### Study measures

Baseline demographic and clinical characteristics including age, gender, race, ethnicity, payer, and Charlson Comorbidity Index (CCI) were measured during the baseline period and reported at index. Comorbidities, procedures, medication use, healthcare resource utilization (HCRU), costs, and charges were measured during the follow-up period.

The clinical characteristics encompassed diseases and conditions included in the CCI, along with the prevalence of additional comorbidities identified through ICD-9 and ICD-10 diagnosis claims (refer to Table E1 in the Online Supplement). Other comorbidities, based on ICD-9 and ICD-10 diagnosis claims, included respiratory conditions or symptoms such as respiratory failure, asthma, pneumonia, and shortness of breath, as well as hypertension, hyperlipidemia, psychiatric conditions (e.g., anxiety and depression), obesity, general weakness, and fatigue. The ICD-9 and ICD-10 codes used to define “other respiratory conditions” were distinct from those used to define ‘rare respiratory diseases” and did not affect eligibility criteria.

Procedures were defined via CPT, HCPCS, ICD-9, and ICD-10 procedure codes (See Table E2 in the Online Supplement) and included bronchoscopy, COVID-19 testing, imaging procedures of the chest, bronchoalveolar lavage (BAL), total lung lavage, mobility assistance devices, oxygen treatments, pulmonary function tests, spirometry, and thoracoscopy. Medications were defined via USC and NDC codes and were grouped into the following categories: antibiotics, inhaled beta agonists, inhaled anticholinergics, inhaled bronchial combination therapies, inhaled steroids, sargramostim, respiratory biologic therapies (i.e., IL-5, anti-IgE, and TSLP biologics), and rituximab.

HCRU metrics included the number of inpatient visits, outpatient visits, emergency room visits, inpatient hospital lengths of stay reported as per patient per year (PPPY). Healthcare interactions (i.e., inpatient visits, outpatient visits, etc.,) were defined via CPT codes, HCPCPs codes, and Place of Service (POS) claims and institutional claims where the healthcare setting was described.

### Statistical analysis

Clinical characteristics, procedures, and therapy use outcomes measured during the pre-index and post-index periods were reported as frequencies. HCRU metric outcomes measured during the post-index period were reported using means, standard deviations, medians, and interquartile ranges (IQR). *P* values for comparisons between clinical characteristics, procedures, therapy use, HCRU mean metrics, and cost among the PAP cohort and control cohort were calculated via independent T tests.

## Results

### Baseline demographic and clinical characteristics

After applying inclusion criteria, this study identified a sample of 2312 PAP patients and 9,247 control patients, which were well balanced after matching with no significant differences in age (*P* = 0.99705) or gender (*P* = 0.99745) (Table 1). PAP patients were mostly (68%) female and had an average age of 61 ± 17.75 years with ~ 75% of patients ≥ 45 years. Over half (63%) of PAP patients were white (non-Hispanic), 9% were Hispanic, and 6% Black or African American. Medicare coverage was reported for 36% of PAP patients and commercial coverage for 30%.

**Table 1:** Baseline characteristics

Patient demographics	PAP cohort (*N* = 2312)		Matched control cohort (*N* = 9247)		*P* value
	N	%	N	%	
*Age*					0.99705
Mean (SD)	61	17.75	61	17.75	
*Gender*					0.99745
Male	740	32	2960	32	
Female	1572	68	6287	68	
*Patient race & ethnicity*					
White (non-hispanic)	1461	63	5839	63	
White (hispanic)	211	9	743	8	
Black or African American	139	6	435	5	
Asian	42	2	195	2	
American Indian or Alaska Native	0	0	0	0	
Native Hawaiian or Other Pacific Islander	5	0	22	0	
Unknown Race	454	20	2013	22	
*Patient payer coverage*					
Commercial	759	33	2636	29	< 0.001
FFS medicaid	110	5	280	3	< 0.001
Managed medicaid	348	15	587	6	< 0.001
Medicare	831	36	2114	23	< 0.001
Other payer	46	2	295	3	< 0.005
Unknown payer	218	9	3335	36	< 0.001
*Charlson comorbidity index, n (%)*					
Score, mean (SD)	1.44	1.96	0.41	1.11	
COPD	726	31	713	8	< 0.001
Diabetes	477	21	811	9	< 0.001
Renal disease	296	13	403	4	< 0.001
Congestive heart failure	307	13	304	3	< 0.001
Diabetes with complications	275	12	377	4	< 0.001
Non-metastatic malignancies	264	11	457	5	< 0.001
Peripheral vascular disease	253	11	311	3	< 0.001
Cerebrovascular disease	187	8	273	3	< 0.001
Mild liver disease	165	7	174	2	< 0.001
Rheumatologic disease	134	6	118	1	< 0.001
Myocardial infarction	86	4	113	1	< 0.001
Dementia	46	2	134	1	< 0.05
Metastatic solid tumor	40	2	60	1	< 0.001
Hemiplegia paraplegia	34	1	21	0	< 0.001
Peptic ulcer disease	27	1	38	0	< 0.001
AIDS	23	1	18	0	< 0.001
Moderate or severe liver disease	8	0	16	0	< 0.05

Mean CCI was significantly higher for PAP patients with a score of 1.44 ± 1.96 compared with 0.41 ± 1.11 for the control cohort (*P* < 0.05) (Table 1). At baseline, 31% of PAP patients had a reported diagnosis of COPD compared with 8% of the control cohort (*P* < 0.001). Significantly higher rates of diabetes (21% vs. 9%; *P* < 0.001), renal disease (13% vs. 4%; *P* < 0.001), congestive heart failure (13% vs. 3%; *P* < 0.001), non-metastatic malignancies (11% vs. 5%; *P* < 0.001) and peripheral vascular disease (11% vs. 3%; *P* < 0.001) were observed in the PAP cohort compared with the control cohort (Table 1).

The ML model, which was not used in the primary analysis of healthcare burden and thus not included in Table 1, identified an additional 4147 likely undiagnosed PAP patients.

### Comorbidities during follow-up

Diagnosis rates for other respiratory conditions (61% vs. 19%; *P* < 0.001), hypertension (49% vs. 22%; *P* < 0.001), hyperlipidemia (36% vs. 17%; *P* < 0.001), psychiatric conditions (29% vs. 11%; *P* < 0.001), obesity (19% vs. 7%; *P* < 0.001), and weakness or fatigue (17% vs. 7%; *P* < 0.001) during the follow-up period were all significantly higher for the PAP cohort compared with the control cohort (Table 2).

**Table 2:** Comorbidities, procedures, and medication use during follow-up period

	PAP cohort (*N* = 2312)		Matched control cohort (*N* = 9247)		*P* value
	N	%	N	%	
*Comorbidities*					
Other respiratory conditions	1415	61	1724	19	< 0.001
Hypertension	1132	49	2075	22	< 0.001
Hyperlipidemia	828	36	1561	17	< 0.001
Psychiatric conditions	659	29	1039	11	< 0.001
Obesity	430	19	614	7	< 0.001
Weakness or fatigue	383	17	603	7	< 0.001
*Procedures*					
Imaging of chest	932	40	932	10	< 0.001
Oxygen treatment	339	15	114	1	< 0.001
Pulmonary function tests	267	12	69	1	< 0.001
COVID-19 testing	279	12	401	4	< 0.001
Spirometry	196	8	91	1	< 0.001
Bronchoscopy	178	8	11	0	< 0.001
Mobility assistance	144	6	177	2	< 0.001
BAL	110	5	5	0	< 0.001
Total lung lavage	53	2	0	0	< 0.001
Thoracoscopy	18	1	3	0	< 0.001
*Medications*					
Antibiotics	457	20	767	8	< 0.001
Inhaled beta agonists	341	15	399	4	< 0.001
Inhaled anticholinergics	155	7	118	1	< 0.001
Inhaled bronchial combination therapies	139	6	130	1	< 0.001
Inhaled steroids	62	3	52	1	< 0.001
Sargramostim	26	1	0	0	< 0.001
Other respiratory therapies	9	0	3	0	< 0.001
Respiratory biologics	4	0	1	0	< 0.001
Rituximab	6	0	6	0	< 0.001

### Procedures during follow-up

During the follow-up period, 40% of PAP patients had a claim for imaging of the chest compared with 10% of the matched controls (*P* < 0.001). Procedure rates for oxygen treatments (15% vs. 1%; *P* < 0.001), pulmonary function tests (12% vs 1%; *P* < 0.001), COVID-19 testing (12% vs. 4%; *P* < 0.001), spirometry (8% vs. 1%; *P* < 0.001), bronchoscopy (8% vs. 0%; *P* < 0.001), mobility assistance devices (6% vs. 2%; *P* < 0.001), BAL (5% vs. 0%; *P* < 0.001), total lung lavage (2% vs. 0%; *P* < 0.001), and thoracoscopy (1% vs. 0%; *P* < 0.001) were significantly higher among PAP patients during the follow-up period (Table 2).

### Medication use during follow-up

More than twice the percentage of PAP patients (20%) utilized significantly higher rates of antibiotics (*P* < 0.001) during the follow-up period compared with the control cohort (8%). Inhaled beta agonists (15% vs. 4%; *P* < 0.001), inhaled anticholinergics (7% vs. 1%; *P* < 0.001), inhaled bronchial combination therapies (6% vs. 1%; *P* < 0.001), and inhaled steroids (3% vs. 1%; *P* < 0.001) were also used by a significantly higher percentage of PAP patients when compared to the control cohort. The use of sargramostim, other respiratory therapies, respiratory biologics, and rituximab was also higher among PAP patients compared with control patients (Table 2).

### Healthcare resource utilization during follow-up

Mean calculations for HCRU metrics revealed higher rates of healthcare interaction across each setting among PAP patients (Table 3). Outpatient visits occurred in 87% of the PAP cohort compared with 56% of the control cohort. Utilization rates were also higher for PAP patients for emergency room visits (35% vs. 14%), and inpatient visits (20% vs. 5%).

**Table 3:** Healthcare resource utilization, costs, and charges during follow-up period

	PAP cohort (*N* = 2312)		Matched control cohort (*N* = 9247)		*P* value
	Mean	SD	Mean	SD	
*Outpatient visits*					
Patients with outpatient visit, n (%)	2002	87%	5142	56%	
Number of outpatient visits, mean (SD)	10.7	10.9	4.0	7	< 0.001
*Emergency room visits*					
Patients with emergency room visit, n (%)	802	35%	1260	14%	
Number of emergency room visits, mean (SD)	1.0	2.3	0.29	1	< 0.001
*Inpatient Visits*					
Patients with inpatient visit, n (%)	467	20%	424	5%	
Number of inpatient visits, mean (SD)	2.3	7.8	0.38	2.5	< 0.001
Length of stay*					
Length of stay (Days), mean (SD)	2.8	7.6	0.56	2.9	< 0.001
*Pharmacy costs*					
Plan-paid, MEAN (SD)	$3685.45	$17,531.58	$839.44	$6457.24	< 0.001
Patient out-of-pocket, mean (SD)	$345.50	$1276.07	$153.24	$836.45	< 0.001
*Non-pharmacy charges*					
Non-pharmacy charges, mean (SD)	$71,672.64	$226,117.54	$14,655.91	$74,791.03	< 0.001

*Among patients with inpatient hospitalizations

Outpatient visits (10.7 ± 10.9, vs. 4.0 ± 7.0; *P* < 0.001), emergency room visits (1.0 ± 2.3, vs. 0.29 ± 1.0; *P* < 0.001), inpatient visits (2.3 ± 7.8, vs. 0.38 ± 2.5; *P* < 0.001), and inpatient hospital length of stay (2.8 days ± 7.6 days, vs. 0.56 days ± 2.9 days; *P* < 0.001) were significantly higher among PAP vs. control patients, respectively.

### Costs and charges during follow-up

Mean plan-paid pharmacy costs during the 1-year follow-up period were significantly higher for PAP patients at $3685.45 (± $17,531.58; *P* < 0.001) compared with $839.44 (± $6,457.24) for control patients (Table 3). Out-of-pocket pharmacy costs were also significantly higher for PAP patients ($345.50 ± $1276.07; *P* < 0.001) compared with the control cohort ($153.24 ± $836.45). Average charges during the follow-up period were significantly higher for the PAP cohort ($71,672.64 ± $226,117.54; *P* < 0.001) compared with the control cohort ($14,655.91 ± $74,791.03) as well.

## Discussion

This analysis yielded 2312 confirmed PAP patients and 9247 matched controls, comprising the most extensive dataset analyzed to date in the United States. The identified PAP cohort in this study constitutes only a sample of total diagnosed PAP patients, as some diagnosed patients were excluded from the analysis if they did not have evidence of claims activity during the 12 months prior to, and 12 months after the index date. In addition, because of the index date window, this analysis reflects data from a snapshot in time at any point during these PAP patients’ disease course and does not necessarily represent their healthcare utilization at the time of diagnosis. We compared demographics, comorbidities, procedures, therapies, healthcare utilization, and costs between the identified sample of PAP patients and controls. In both the PAP patient cohort and the matched-control cohort, there were more females than males who were older, with more than half being older than 55 years old at the time of the study. Additionally, patients in both cohorts were covered primarily by Medicare, Commercial, and Medicaid insurance plans. PAP patients had increased rates of comorbidities, procedures, imaging, healthcare interactions including outpatient visits, emergency room visits, inpatient visits, pharmaceutical and non-pharmaceutical costs, and therapy use compared with matched control patients.

Results of this study are complementary to a previous epidemiologic study performed on a smaller subset of 249 confirmed PAP patients in the U.S. [9] as well as a prior study examining the cost of hospital admissions in PAP patients based on a U.S. claims database [21]. Our study validates previous findings that demonstrated increased rates of comorbidities (e.g., COPD) as well as increased healthcare utilization and costs due to increased outpatient visits, emergency room visits, and longer inpatient hospital stays in PAP patients compared with matched controls. Annual per-patient healthcare costs were also higher due to increased costs of inpatient visits, outpatient visits, and prescriptions. Interestingly, while we had more females than males in our cohorts, we did not see a gender difference in any of these parameters. This gender distribution differed from a retrospective meta-analysis conducted by Seymour and colleagues [2] that found a male predominance in PAP, which was also seen by Inoue and colleagues [10] in their Japanese cohort. One possible contributor to this observation is that, in the U.S., women are more likely than men to use the healthcare system [22], which may have skewed claims data toward a higher female to male ratio. In addition, more recently published studies on PAP patient cohorts and the U.S.-based PAP patient registry suggest that the gender distribution is more evenly divided [9, 23]. Thus, more studies need to be performed to see if a true gender predilection exists within PAP patients.

Accurately diagnosing and treating patients with this rare lung disease is challenging due to nonspecific symptoms, limited access to testing, and lack of approved therapies. To explore the possibility of an undiagnosed cohort of PAP patients, we utilized IPM.ai’s longitudinal claims dataset to develop a ML model to investigate the number of patients who have not yet been diagnosed with PAP but displayed strong indicators of the disease. Our ML model identified an additional 4147 patients from the ~ 300 million individuals in the real-world claims dataset and relied heavily on unique claims patterns that align with the medical history of diagnosed PAP patients. The results of the ML model suggest there may be thousands of potentially undiagnosed PAP patients across the U.S. who have not received proper care. These patients may warrant further investigation and represent an opportunity for improvement in the timely and accurate diagnosis of this syndrome. This is especially true for patients with autoimmune PAP, where there is a highly sensitive and specific simple serum test available for GM-CSF autoantibodies that can reduce the cost and morbidity associated with more invasive testing such as lung biopsies and is critical in making a definitive diagnosis of autoimmune PAP.

Some potential limitations of this study should be noted. In general, there are limitations with utilizing a claims dataset since it is dependent on professional ICD coding. Sometimes coding can be inaccurate, and the diagnosis can be missing. Another limitation of the study is that a portion of the data collection occurred during the COVID-19 pandemic and may have underestimated some of the results. There were delays and inability to get necessary healthcare, especially for chronic medical conditions, due to pandemic-related fears, financial burdens, and hospital resources being reallocated to treat patients with COVID-19 [24–28]. Consequently, outpatient visits, procedures like bronchoscopies done to aid in diagnosis, and therapies like whole lung lavages done for management of PAP, may have been reduced and/or delayed. The pandemic may also have impacted claims data collection associated with non-COVID-related conditions and symptoms and may have affected our ML model, which heavily relied on previous diagnoses of pulmonary diseases, abnormal pulmonary findings, and common procedures seen and used in PAP patients to identify and characterize an undiagnosed cohort. Lastly, an additional limitation in this study is the inability to distinguish the different types of PAP syndromes based on ICD coding alone. Although the vast majority of PAP patients are autoimmune PAP patients, patients with different PAP syndromes would be expected to have different levels of comorbidities and healthcare utilization.

Nevertheless, this study represents the largest dataset of PAP patients and matched controls to be analyzed to date and clearly shows that PAP patients have a significant healthcare burden of disease compared to non-PAP patients. They have increased comorbidities, healthcare resource utilization, and pharmaceutical and non-pharmaceutical costs. This is largely due to knowledge gaps and poor access to testing for proper diagnosis as well as current therapies being limited to symptom management and ineffective with regard to modifying the disease itself. These rare lung disease patients need increased access to testing and effective treatments that have the potential to reduce the burdens experienced by both the PAP patients themselves and the healthcare system.

## Conclusion

This study represents the largest cohort of PAP patients and matched controls to be analyzed to date and demonstrates that PAP patients have a large incremental burden of disease vs. non-PAP patients. Patients with PAP experience higher healthcare resource utilization, pharmaceutical and non-pharmaceutical costs, comorbidities, procedures, and therapy use, highlighting a significant unmet need in this rare disease patient population.

## Supplementary Information

Additional file 1.

## Data Availability

The data that support the findings of this study are available from IPM.ai but restrictions apply to the availability of these data, which were used under license for the current study, and so are not publicly available. Data are, however, available from the authors upon reasonable request and with permission of IPM.ai and Savara Inc.
