# Supplementary material for: The healthcare burden of pulmonary alveolar proteinosis (PAP)
Source: Orphanet J Rare Dis. 2025 Feb 14;20:73. doi: 10.1186/s13023-024-03478-2 (PMC11829527; doi:10.1186/s13023-024-03478-2)
Supplement: Supplementary file 1 — Additional file 1. [file 13023_2024_3478_MOESM1_ESM.docx]

**Supplementary Appendix**

Lee E, Ataya A, McCarthy C, et al. The Healthcare Burden of Pulmonary Alveolar Proteinosis (PAP)

**Content**

[Table E1. ICD-10 and ICD-9 Codes 2](#_Toc159851142)

[Table E2. CPT, HCPCS, ICD-9 Procedure, and ICD-10 Procedure Codes 12](#_Toc159851143)

# Table E1. ICD-10 and ICD-9 Codes

| **ICD-10 and ICD-9 Codes Used to Define ‘Rare Respiratory Diseases’** | |
| --- | --- |
| **ICD-10 Code** | **Code Description** |
| J82 | Pulmonary eosinophilia, not elsewhere classified |
| J82.81 | Chronic eosinophilic pneumonia |
| J82.82 | Acute eosinophilic pneumonia |
| J82.89 | Other pulmonary eosinophilia, not elsewhere classified |
| J84.02 | Pulmonary alveolar microlithiasis |
| J84.03 | Idiopathic pulmonary hemosiderosis |
| J84.09 | Other alveolar and parieto-alveolar conditions |
| J84.11 | Idiopathic interstitial pneumonia |
| J84.111 | Idiopathic interstitial pneumonia, not otherwise specified |
| J84.112 | Idiopathic pulmonary fibrosis |
| J84.113 | Idiopathic non-specific interstitial pneumonitis |
| J84.114 | Acute interstitial pneumonitis |
| J84.115 | Respiratory bronchiolitis interstitial lung disease |
| J84.116 | Cryptogenic organizing pneumonia |
| J84.117 | Desquamative interstitial pneumonia |
| J84.17 | Other interstitial pulmonary diseases with fibrosis in diseases classified elsewhere |
| J84.170 | Interstitial lung disease with progressive fibrotic phenotype in diseases classified elsewhere |
| J84.178 | Other interstitial pulmonary diseases with fibrosis in diseases classified elsewhere |
| J84.2 | Lymphoid interstitial pneumonia |
| J84.81 | Lymphangioleiomyomatosis |
| J84.82 | Adult pulmonary Langerhans cell histiocytosis |
| J84.83 | Surfactant mutations of the lung |
| J84.841 | Neuroendocrine cell hyperplasia of infancy |
| J84.842 | Pulmonary interstitial glycogenosis |
| J84.843 | Alveolar capillary dysplasia with vein misalignment |
| J84.848 | Other interstitial lung diseases of childhood |
| J84.9 | Interstitial pulmonary disease, unspecified |
| J98.2 | Interstitial emphysema |
| **ICD-9 Code** | **Code Description** |
| 515 | Postinflammatory pulmonary fibrosis |
| 516.1 | Idiopathic pulmonary hemosiderosis |
| 516.2 | Pulmonary alveolar microlithiasis |
| 516.30 | Idiopathic interstitial pneumonia, not otherwise specified |
| 516.31 | Idiopathic pulmonary fibrosis |
| 516.32 | Idiopathic non-specific interstitial pneumonitis |
| 516.33 | Acute interstitial pneumonitis |
| 516.34 | Respiratory bronchiolitis interstitial lung disease |
| 516.35 | Idiopathic lymphoid interstitial pneumonia |
| 516.36 | Cryptogenic organizing pneumonia |
| 516.37 | Desquamative interstitial pneumonia |
| 516.4 | Lymphangioleiomyomatosis |
| 516.5 | Adult pulmonary Langerhans cell histiocytosis |
| 516.61 | Neuroendocrine cell hyperplasia of infancy |
| 516.62 | Pulmonary interstitial glycogenosis |
| 516.63 | Surfactant mutations of the lung |
| 516.64 | Alveolar capillary dysplasia with vein misalignment |
| 516.69 | Other interstitial lung diseases of childhood |
| 516.8 | Other specified alveolar and parietoalveolar pneumonopathies |
| 516.9 | Unspecified alveolar and parietoalveolar pneumonopathy |
| 518.1 | Interstitial emphysema |
| 518.3 | Pulmonary eosinophilia |
| **ICD-10 and ICD-9 Codes Used to Define ‘Other Respiratory Conditions’** | |
| **ICD-10 Code** | **Code Description** |
| J06.9 | Acute upper respiratory infection, unspecified |
| J18.9 | Pneumonia, unspecified organism |
| J20 | Acute bronchitis |
| J20.0 | Acute bronchitis due to Mycoplasma pneumoniae |
| J20.1 | Acute bronchitis due to Hemophilus influenzae |
| J20.2 | Acute bronchitis due to streptococcus |
| J20.3 | Acute bronchitis due to coxsackievirus |
| J20.4 | Acute bronchitis due to parainfluenza virus |
| J20.5 | Acute bronchitis due to respiratory syncytial virus |
| J20.6 | Acute bronchitis due to rhinovirus |
| J20.7 | Acute bronchitis due to echovirus |
| J20.8 | Acute bronchitis due to other specified organisms |
| J20.9 | Acute bronchitis, unspecified |
| J40 | Bronchitis, not specified as acute or chronic |
| J41 | Simple and mucopurulent chronic bronchitis |
| J41.0 | Simple chronic bronchitis |
| J41.1 | Mucopurulent chronic bronchitis |
| J41.8 | Mixed simple and mucopurulent chronic bronchitis |
| J42 | Unspecified chronic bronchitis |
| J43 | Emphysema |
| J43.0 | Unilateral pulmonary emphysema [MacLeod's syndrome] |
| J43.1 | Panlobular emphysema |
| J43.2 | Centrilobular emphysema |
| J43.8 | Other emphysema |
| J43.9 | Emphysema, unspecified |
| J44.0 | Chronic obstructive pulmonary disease with (acute) lower respiratory infection |
| J44.1 | Chronic obstructive pulmonary disease with (acute) exacerbation |
| J44.9 | Chronic obstructive pulmonary disease, unspecified |
| J45 | Asthma |
| J45.2 | Mild intermittent asthma |
| J45.20 | Mild intermittent asthma, uncomplicated |
| J45.21 | Mild intermittent asthma with (acute) exacerbation |
| J45.22 | Mild intermittent asthma with status asthmaticus |
| J45.3 | Mild persistent asthma |
| J45.30 | Mild persistent asthma, uncomplicated |
| J45.31 | Mild persistent asthma with (acute) exacerbation |
| J45.32 | Mild persistent asthma with status asthmaticus |
| J45.4 | Moderate persistent asthma |
| J45.40 | Moderate persistent asthma, uncomplicated |
| J45.41 | Moderate persistent asthma with (acute) exacerbation |
| J45.42 | Moderate persistent asthma with status asthmaticus |
| J45.5 | Severe persistent asthma |
| J45.50 | Severe persistent asthma, uncomplicated |
| J45.51 | Severe persistent asthma with (acute) exacerbation |
| J45.52 | Severe persistent asthma with status asthmaticus |
| J45.9 | Other and unspecified asthma |
| J45.90 | Unspecified asthma |
| J45.901 | Unspecified asthma with (acute) exacerbation |
| J45.902 | Unspecified asthma with status asthmaticus |
| J45.909 | Unspecified asthma, uncomplicated |
| J45.99 | Other asthma |
| J45.991 | Cough variant asthma |
| J45.998 | Other asthma |
| J47 | Bronchiectasis |
| J47.0 | Bronchiectasis with acute lower respiratory infection |
| J47.1 | Bronchiectasis with (acute) exacerbation |
| J47.9 | Bronchiectasis, uncomplicated |
| J68.0 | Bronchitis and pneumonitis due to chemicals, gases, fumes and vapors |
| J84.10 | Pulmonary fibrosis, unspecified |
| J96 | Respiratory failure, not elsewhere classified |
| J96.0 | Acute respiratory failure |
| J96.00 | Acute respiratory failure, unspecified whether with hypoxia or hypercapnia |
| J96.01 | Acute respiratory failure with hypoxia |
| J96.02 | Acute respiratory failure with hypercapnia |
| J96.1 | Chronic respiratory failure |
| J96.10 | Chronic respiratory failure, unspecified whether with hypoxia or hypercapnia |
| J96.11 | Chronic respiratory failure with hypoxia |
| J96.12 | Chronic respiratory failure with hypercapnia |
| J96.2 | Acute and chronic respiratory failure |
| J96.20 | Acute and chronic respiratory failure, unspecified whether with hypoxia or hypercapnia |
| J96.21 | Acute and chronic respiratory failure with hypoxia |
| J96.22 | Acute and chronic respiratory failure with hypercapnia |
| J96.9 | Respiratory failure, unspecified |
| J96.90 | Respiratory failure, unspecified, unspecified whether with hypoxia or hypercapnia |
| J96.91 | Respiratory failure, unspecified with hypoxia |
| J96.92 | Respiratory failure, unspecified with hypercapnia |
| J98.2 | Interstitial emphysema |
| J98.3 | Compensatory emphysema |
| J98.4 | Other disorders of lung |
| P25 | Interstitial emphysema and related conditions originating in the perinatal period |
| P25.0 | Interstitial emphysema originating in the perinatal period |
| P25.8 | Other conditions related to interstitial emphysema originating in the perinatal period |
| Q33.4 | Congenital bronchiectasis |
| R04.2 | Hemoptysis |
| R05 | Cough |
| R06.0 | Dyspnea |
| R06.00 | Dyspnea, unspecified |
| R06.02 | Shortness of breath |
| R06.09 | Other forms of dyspnea |
| R09.3 | Abnormal sputum |
| R91.8 | Other nonspecific abnormal finding of lung field |
| T79.7 | Traumatic subcutaneous emphysema |
| T79.7XXA | Traumatic subcutaneous emphysema, initial encounter |
| T79.7XXD | Traumatic subcutaneous emphysema, subsequent encounter |
| T79.7XXS | Traumatic subcutaneous emphysema, sequela |
| T81.82 | Emphysema (subcutaneous) resulting from a procedure |
| T81.82XA | Emphysema (subcutaneous) resulting from a procedure, initial encounter |
| T81.82XD | Emphysema (subcutaneous) resulting from a procedure, subsequent encounter |
| T81.82XS | Emphysema (subcutaneous) resulting from a procedure, sequela |
| U07.1 | COVID-19 |
| U09 | Post COVID-19 condition |
| U09.9 | Post COVID-19 condition, unspecified |
| V81.3 | Screening for chronic bronchitis and emphysema |
| Z86.16 | Personal history of COVID-19 |
| **ICD-9 Code** | **Code Description** |
| 490 | Bronchitis, not specified as acute or chronic |
| 491.0 | Simple chronic bronchitis |
| 491.1 | Mucopurulent chronic bronchitis |
| 491.20 | Obstructive chronic bronchitis without exacerbation |
| 491.21 | Obstructive chronic bronchitis with (acute) exacerbation |
| 491.22 | Obstructive chronic bronchitis with acute bronchitis |
| 491.8 | Other chronic bronchitis |
| 491.9 | Unspecified chronic bronchitis |
| 492.0 | Emphysematous bleb |
| 492.8 | Other emphysema |
| 493.00 | Extrinsic asthma, unspecified |
| 493.01 | Extrinsic asthma with status asthmaticus |
| 493.02 | Extrinsic asthma with (acute) exacerbation |
| 493.10 | Intrinsic asthma, unspecified |
| 493.11 | Intrinsic asthma with status asthmaticus |
| 493.12 | Intrinsic asthma with (acute) exacerbation |
| 493.20 | Chronic obstructive asthma, unspecified |
| 493.21 | Chronic obstructive asthma with status asthmaticus |
| 493.22 | Chronic obstructive asthma with (acute) exacerbation |
| 493.82 | Cough variant asthma |
| 493.90 | Asthma, unspecified type, unspecified |
| 493.91 | Asthma, unspecified type, with status asthmaticus |
| 493.92 | Asthma, unspecified type, with (acute) exacerbation |
| 494.1 | Bronchiectasis with acute exacerbation |
| 496 | Chronic airway obstruction, not elsewhere classified |
| 518.1 | Interstitial emphysema |
| 518.2 | Compensatory emphysema |
| 518.51 | Acute respiratory failure following trauma and surgery |
| 518.53 | Acute and chronic respiratory failure following trauma and surgery |
| 518.81 | Acute respiratory failure |
| 518.83 | Chronic respiratory failure |
| 518.84 | Acute and chronic respiratory failure |
| 748.61 | Congenital bronchiectasis |
| 770.2 | Interstitial emphysema and related conditions |
| 786.05 | Shortness of breath |
| 786.09 | Other respiratory abnormalities |
| 786.30 | Hemoptysis, unspecified |
| 786.39 | Other hemoptysis |
| 786.4 | Abnormal sputum |
| 958.7 | Traumatic subcutaneous emphysema |
| 998.81 | Emphysema (subcutaneous) (surgical) resulting from procedure |
| **ICD-10 and ICD-9 Codes Used to Define ‘Hypertension’** | |
| **ICD-10 Code** | **Code Description** |
| I10 | Essential (primary) hypertension |
| I15.9 | Secondary hypertension, unspecified |
| **ICD-9 Code** | **Code Description** |
| 401.0 | Malignant essential hypertension |
| 401.1 | Benign essential hypertension |
| 401.9 | Unspecified essential hypertension |
| 405.99 | Other unspecified secondary hypertension |
| **ICD-10 and ICD-9 Codes Used to Define ‘Hyperlipidemia’** | |
| **ICD-10 Code** | **Code Description** |
| E78.2 | Mixed hyperlipidemia |
| E78.4 | Other hyperlipidemia |
| E78.49 | Other hyperlipidemia |
| E78.5 | Hyperlipidemia, unspecified |
| **ICD-9 Code** | **Code Description** |
| 272.2 | Mixed hyperlipidemia |
| 272.4 | Other and unspecified hyperlipidemia |
| **ICD-10 and ICD-9 Codes Used to Define ‘Psychiatric Conditions’** | |
| **ICD-10 Code** | **Code Description** |
| F06.31 | Mood disorder due to known physiological condition with depressive features |
| F25.1 | Schizoaffective disorder, depressive type |
| F32.0 | Major depressive disorder, single episode, mild |
| F32.1 | Major depressive disorder, single episode, moderate |
| F32.2 | Major depressive disorder, single episode, severe without psychotic features |
| F32.3 | Major depressive disorder, single episode, severe with psychotic features |
| F32.4 | Major depressive disorder, single episode, in partial remission |
| F32.5 | Major depressive disorder, single episode, in full remission |
| F32.89 | Other specified depressive episodes |
| F32.9 | Major depressive disorder, single episode, unspecified |
| F33.0 | Major depressive disorder, recurrent, mild |
| F33.1 | Major depressive disorder, recurrent, moderate |
| F33.2 | Major depressive disorder, recurrent severe without psychotic features |
| F33.3 | Major depressive disorder, recurrent, severe with psychotic symptoms |
| F33.40 | Major depressive disorder, recurrent, in remission, unspecified |
| F33.41 | Major depressive disorder, recurrent, in partial remission |
| F33.42 | Major depressive disorder, recurrent, in full remission |
| F33.8 | Other recurrent depressive disorders |
| F33.9 | Major depressive disorder, recurrent, unspecified |
| F41.0 | Panic disorder [episodic paroxysmal anxiety] |
| F41.1 | Generalized anxiety disorder |
| F41.8 | Other specified anxiety disorders |
| F41.9 | Anxiety disorder, unspecified |
| **ICD-9 Code** | **Code Description** |
| 298.0 | Depressive type psychosis |
| 293.83 | Mood disorder in conditions classified elsewhere |
| 295.70 | Schizoaffective disorder, unspecified |
| 296.20 | Major depressive affective disorder, single episode, unspecified |
| 296.21 | Major depressive affective disorder, single episode, mild |
| 296.22 | Major depressive affective disorder, single episode, moderate |
| 296.23 | Major depressive affective disorder, single episode, severe, without mention of psychotic behavior |
| 296.24 | Major depressive affective disorder, single episode, severe, specified as with psychotic behavior |
| 296.25 | Major depressive affective disorder, single episode, in partial or unspecified remission |
| 296.26 | Major depressive affective disorder, single episode, in full remission |
| 296.30 | Major depressive affective disorder, recurrent episode, unspecified |
| 296.31 | Major depressive affective disorder, recurrent episode, mild |
| 296.32 | Major depressive affective disorder, recurrent episode, moderate |
| 296.33 | Major depressive affective disorder, recurrent episode, severe, without mention of psychotic behavior |
| 296.34 | Major depressive affective disorder, recurrent episode, severe, specified as with psychotic behavior |
| 296.35 | Major depressive affective disorder, recurrent episode, in partial or unspecified remission |
| 296.36 | Major depressive affective disorder, recurrent episode, in full remission |
| 296.99 | Other specified episodic mood disorder |
| 300.00 | Anxiety state, unspecified |
| 300.01 | Panic disorder without agoraphobia |
| 300.02 | Generalized anxiety disorder |
| 300.09 | Other anxiety states |
| **ICD-10 Codes Used to Define ‘Obesity’** | |
| **ICD-10 Code** | **Code Description** |
| E66.01 | Morbid (severe) obesity due to excess calories |
| E66.09 | Other obesity due to excess calories |
| E66.2 | Morbid (severe) obesity with alveolar hypoventilation |
| E66.8 | Other obesity |
| E66.9 | Obesity, unspecified |
| **ICD-10 Codes Used to Define ‘Weakness and Fatigue’** | |
| **ICD-10 Code** | **Code Description** |
| R53 | Malaise and fatigue |
| R53.1 | Weakness |
| R53.8 | Other malaise and fatigue |
| R53.82 | Chronic fatigue, unspecified |
| R53.83 | Other fatigue |

# Table E2. CPT, HCPCS, ICD-9 Procedure, and ICD-10 Procedure Codes

| **Procedure Codes Used to Define ‘Imaging of Chest’** | |
| --- | --- |
| **Procedure Code** | **Code Description** |
| 71010 | Radiologic examination, chest; single view, frontal |
| 71010 | Radiologic examination, chest; single view, frontal |
| 71015 | Radiologic examination, chest; stereo, frontal |
| 71020 | Radiologic examination, chest, 2 views, frontal and lateral; |
| 71021 | Radiologic examination, chest, 2 views, frontal and lateral; with apical lordotic procedure |
| 71022 | Radiologic examination, chest, 2 views, frontal and lateral; with oblique projections |
| 71023 | Radiologic examination, chest, 2 views, frontal and lateral; with fluoroscopy |
| 71030 | Radiologic examination, chest, complete, minimum of 4 views; |
| 71034 | Radiologic examination, chest, complete, minimum of 4 views; with fluoroscopy |
| 71035 | Radiologic examination, chest, special views (eg, lateral decubitus, Bucky studies) |
| 71045 | Radiologic examination, chest; single view |
| 71046 | Radiologic examination, chest; 2 views |
| 71047 | Radiologic examination, chest; 3 views |
| 71048 | Radiologic examination, chest; 4 or more views |
| 71101 | Radiologic examination, ribs, unilateral; including posteroanterior chest, minimum of 3 views |
| 71111 | Radiologic examination, ribs, bilateral; including posteroanterior chest, minimum of 4 views |
| 74022 | Radiologic examination, complete acute abdomen series, including 2 or more views of the abdomen (eg, supine, erect, decubitus), and a single view chest |
| 74220 | Radiologic examination, esophagus, including scout chest radiograph(s) and delayed image(s), when performed; single-contrast (eg, barium) study |
| 74221 | Radiologic examination, esophagus, including scout chest radiograph(s) and delayed image(s), when performed; double-contrast (eg, high-density barium and effervescent agent) study |
| 71020 | Radiologic examination, chest, 2 views, frontal and lateral; |
| 71021 | Radiologic examination, chest, 2 views, frontal and lateral; with apical lordotic procedure |
| 71023 | Radiologic examination, chest, 2 views, frontal and lateral; with fluoroscopy |
| 71030 | Radiologic examination, chest, complete, minimum of 4 views; |
| 71035 | Radiologic examination, chest, special views (eg, lateral decubitus, Bucky studies) |
| 71045 | Radiologic examination, chest; single view |
| 71046 | Radiologic examination, chest; 2 views |
| 71047 | Radiologic examination, chest; 3 views |
| 71048 | Radiologic examination, chest; 4 or more views |
| 71250 | Computed tomography, thorax; without contrast material |
| 71260 | Computed tomography, thorax; with contrast material(s) |
| 71270 | Computed tomography, thorax; without contrast material, followed by contrast material(s) and further sections |
| 71271 | Computed Tomography, Thorax, Low Dose For Lung Cancer Screening, Without Contrast Material(S) |
| BH3G | Magnetic Resonance Imaging (MRI) / Subcutaneous Tissue, Thorax |
| BH3GY | Magnetic Resonance Imaging (MRI) / Subcutaneous Tissue, Thorax / Other Contrast |
| BH3GY0 | Magnetic Resonance Imaging (MRI) / Subcutaneous Tissue, Thorax / Other Contrast / Unenhanced and Enhanced |
| BH3GY0Z | Magnetic Resonance Imaging (MRI) of Thorax Subcutaneous Tissue using Other Contrast, Unenhanced and Enhanced |
| BH3GYZ | Magnetic Resonance Imaging (MRI) / Subcutaneous Tissue, Thorax / Other Contrast / None |
| BH3GYZZ | Magnetic Resonance Imaging (MRI) of Thorax Subcutaneous Tissue using Other Contrast |
| BH3GZ | Magnetic Resonance Imaging (MRI) / Subcutaneous Tissue, Thorax / None |
| BH3GZZ | Magnetic Resonance Imaging (MRI) / Subcutaneous Tissue, Thorax / None / None |
| BH3GZZZ | Magnetic Resonance Imaging (MRI) of Thorax Subcutaneous Tissue |
| BP2W | Computerized Tomography (CT Scan) / Thorax |
| BP2W0 | Computerized Tomography (CT Scan) / Thorax / High Osmolar |
| BP2W0Z | Computerized Tomography (CT Scan) / Thorax / High Osmolar / None |
| BP2W0ZZ | Computerized Tomography (CT Scan) of Thorax using High Osmolar Contrast |
| BP2W1 | Computerized Tomography (CT Scan) / Thorax / Low Osmolar |
| BP2W1Z | Computerized Tomography (CT Scan) / Thorax / Low Osmolar / None |
| BP2W1ZZ | Computerized Tomography (CT Scan) of Thorax using Low Osmolar Contrast |
| BP2WY | Computerized Tomography (CT Scan) / Thorax / Other Contrast |
| BP2WYZ | Computerized Tomography (CT Scan) / Thorax / Other Contrast / None |
| BP2WYZZ | Computerized Tomography (CT Scan) of Thorax using Other Contrast |
| BW24 | Computerized Tomography (CT Scan) / Chest and Abdomen |
| BW240 | Computerized Tomography (CT Scan) / Chest and Abdomen / High Osmolar |
| BW2400 | Computerized Tomography (CT Scan) / Chest and Abdomen / High Osmolar / Unenhanced and Enhanced |
| BW2400Z | Computerized Tomography (CT Scan) of Chest and Abdomen using High Osmolar Contrast, Unenhanced and Enhanced |
| BW240Z | Computerized Tomography (CT Scan) / Chest and Abdomen / High Osmolar / None |
| BW240ZZ | Computerized Tomography (CT Scan) of Chest and Abdomen using High Osmolar Contrast |
| BW241 | Computerized Tomography (CT Scan) / Chest and Abdomen / Low Osmolar |
| BW2410 | Computerized Tomography (CT Scan) / Chest and Abdomen / Low Osmolar / Unenhanced and Enhanced |
| BW2410Z | Computerized Tomography (CT Scan) of Chest and Abdomen using Low Osmolar Contrast, Unenhanced and Enhanced |
| BW241Z | Computerized Tomography (CT Scan) / Chest and Abdomen / Low Osmolar / None |
| BW241ZZ | Computerized Tomography (CT Scan) of Chest and Abdomen using Low Osmolar Contrast |
| BW24Y | Computerized Tomography (CT Scan) / Chest and Abdomen / Other Contrast |
| BW24Y0 | Computerized Tomography (CT Scan) / Chest and Abdomen / Other Contrast / Unenhanced and Enhanced |
| BW24Y0Z | Computerized Tomography (CT Scan) of Chest and Abdomen using Other Contrast, Unenhanced and Enhanced |
| BW24YZ | Computerized Tomography (CT Scan) / Chest and Abdomen / Other Contrast / None |
| BW24YZZ | Computerized Tomography (CT Scan) of Chest and Abdomen using Other Contrast |
| BW24Z | Computerized Tomography (CT Scan) / Chest and Abdomen / None |
| BW24ZZ | Computerized Tomography (CT Scan) / Chest and Abdomen / None / None |
| BW24ZZZ | Computerized Tomography (CT Scan) of Chest and Abdomen |
| BW25 | Computerized Tomography (CT Scan) / Chest, Abdomen and Pelvis |
| BW250 | Computerized Tomography (CT Scan) / Chest, Abdomen and Pelvis / High Osmolar |
| BW2500 | Computerized Tomography (CT Scan) / Chest, Abdomen and Pelvis / High Osmolar / Unenhanced and Enhanced |
| BW2500Z | Computerized Tomography (CT Scan) of Chest, Abdomen and Pelvis using High Osmolar Contrast, Unenhanced and Enhanced |
| BW250Z | Computerized Tomography (CT Scan) / Chest, Abdomen and Pelvis / High Osmolar / None |
| BW250ZZ | Computerized Tomography (CT Scan) of Chest, Abdomen and Pelvis using High Osmolar Contrast |
| BW251 | Computerized Tomography (CT Scan) / Chest, Abdomen and Pelvis / Low Osmolar |
| BW2510 | Computerized Tomography (CT Scan) / Chest, Abdomen and Pelvis / Low Osmolar / Unenhanced and Enhanced |
| BW2510Z | Computerized Tomography (CT Scan) of Chest, Abdomen and Pelvis using Low Osmolar Contrast, Unenhanced and Enhanced |
| BW251Z | Computerized Tomography (CT Scan) / Chest, Abdomen and Pelvis / Low Osmolar / None |
| BW251ZZ | Computerized Tomography (CT Scan) of Chest, Abdomen and Pelvis using Low Osmolar Contrast |
| BW25Y | Computerized Tomography (CT Scan) / Chest, Abdomen and Pelvis / Other Contrast |
| BW25Y0 | Computerized Tomography (CT Scan) / Chest, Abdomen and Pelvis / Other Contrast / Unenhanced and Enhanced |
| BW25Y0Z | Computerized Tomography (CT Scan) of Chest, Abdomen and Pelvis using Other Contrast, Unenhanced and Enhanced |
| BW25YZ | Computerized Tomography (CT Scan) / Chest, Abdomen and Pelvis / Other Contrast / None |
| BW25YZZ | Computerized Tomography (CT Scan) of Chest, Abdomen and Pelvis using Other Contrast |
| BW25Z | Computerized Tomography (CT Scan) / Chest, Abdomen and Pelvis / None |
| BW25ZZ | Computerized Tomography (CT Scan) / Chest, Abdomen and Pelvis / None / None |
| BW25ZZZ | Computerized Tomography (CT Scan) of Chest, Abdomen and Pelvis |
| **Procedure Codes Used to Define ‘Oxygen Treatment’** | |
| **Procedure Code** | **Code Description** |
| E0430 | Portable gaseous oxygen system, purchase; includes regulator, flowmeter, humidifier, cannula or mask, and tubing |
| E0431 | Portable gaseous oxygen system, rental; includes portable container, regulator, flowmeter, humidifier, cannula or mask, and tubing |
| K0738 | Portable gaseous oxygen system, rental; home compressor used to fill portable oxygen cylinders; includes portable containers, regulator, flowmeter, humidifier, cannula or mask, and tubing |
| K0741 | Portable gaseous oxygen system, rental, includes portable container, regulator, flowmeter, humidifier, cannula or mask, and tubing, for cluster headaches |
| E1377 | Oxygen concentrator, high humidity system equiv. to 244 cu. ft. |
| E1378 | Oxygen concentrator, high humidity system equiv. to 488 cu. ft. |
| E1379 | Oxygen concentrator, high humidity system equiv. to 732 cu. ft. |
| E1380 | Oxygen concentrator, high humidity system equiv. to 976 cu. ft. |
| E1381 | Oxygen concentrator, high humidity system equiv. to 1220 cu. ft. |
| E1382 | Oxygen concentrator, high humidity system equiv. to 1464 cu. ft. |
| E1383 | Oxygen concentrator, high humidity system equiv. to 1708 cu. ft. |
| E1384 | Oxygen concentrator, high humidity system equiv. to 1952 cu. ft. |
| E1385 | Oxygen concentrator, high humidity system equiv. to over 1952 cu. ft. |
| E1390 | Oxygen concentrator, single delivery port, capable of delivering 85 percent or greater oxygen concentration at the prescribed flow rate |
| E1391 | Oxygen concentrator, dual delivery port, capable of delivering 85 percent or greater oxygen concentration at the prescribed flow rate, each |
| E1392 | Portable oxygen concentrator, rental |
| E1400 | Oxygen concentrator, manufacturer specified maximum flow rate does not exceed two liters per minute, at 85 percent or greater concentration |
| E1401 | Oxygen concentrator, manufacturer specified maximum flow rate greater than two liters per minute, does not exceed three liters per minute, at 85 percent or greater concentration |
| E1402 | Oxygen concentrator, manufacturer specified maximum flow rate greater than three liters per minute, does not exceed four liters per minute, at 85 percent or greater concentration |
| E1403 | Oxygen concentrator, manufacturer specified maximum flow rate greater than four liters per minute, does not exceed five liters per minute, at 85 percent or greater concentration |
| E1404 | Oxygen concentrator, manufacturer specified maximum flow rate greater than five liters per minute, at 85 percent or greater concentration |
| K0671 | Portable oxygen concentrator, rental |
| A0422 | Ambulance (ALS or BLS) oxygen and oxygen supplies, life sustaining situation |
| E0443 | Portable oxygen contents, gaseous, 1 month's supply = 1 unit |
| E0444 | Portable oxygen contents, liquid, 1 month's supply = 1 unit |
| E0447 | Portable oxygen contents, liquid, 1 month's supply = 1 unit, prescribed amount at rest or nighttime exceeds 4 liters per minute (LPM) |
| K0742 | Portable oxygen contents, gaseous, 1 month's supply = 1 unit, for cluster headaches, for initial months supply or to replace used contents |
| E0433 | Portable liquid oxygen system, rental; home liquefier used to fill portable liquid oxygen containers, includes portable containers, regulator, flowmeter, humidifier, cannula or mask and tubing, with or without supply reservoir and contents gauge |
| E0434 | Portable liquid oxygen system, rental; includes portable container, supply reservoir, humidifier, flowmeter, refill adaptor, contents gauge, cannula or mask, and tubing |
| E0435 | Portable liquid oxygen system, purchase; includes portable container, supply reservoir, flowmeter, humidifier, contents gauge, cannula or mask, tubing and refill adaptor |
| E0441 | Stationary oxygen contents, gaseous, 1 month's supply = 1 unit |
| E0442 | Stationary oxygen contents, liquid, 1 month's supply = 1 unit |
| S8120 | Oxygen contents, gaseous, 1 unit equals 1 cubic foot |
| S8121 | Oxygen contents, liquid, 1 unit equals 1 pound |
| **Procedure Codes Used to Define ‘Pulmonary Function Test’** | |
| **Procedure Code** | **Code Description** |
|  |  |
| 94013 | Measurement of lung volumes (ie, functional residual capacity [FRC], forced vital capacity [FVC], and expiratory reserve volume [ERV]) in an infant or child through 2 years of age |
| 94726 | Plethysmography for determination of lung volumes and, when performed, airway resistance |
| 94727 | Gas dilution or washout for determination of lung volumes and, when performed, distribution of ventilation and closing volumes |
| 94729 | Diffusing capacity (eg, carbon monoxide, membrane) (List separately in addition to code for primary procedure) |
| 94618 | Pulmonary stress testing (eg, 6-minute walk test), including measurement of heart rate, oximetry, and oxygen titration, when performed |
| 94620 | Pulmonary stress testing; simple (eg, 6-minute walk test, prolonged exercise test for bronchospasm with pre- and post-spirometry and oximetry) |
| **Procedure Codes Used to Define ‘COVID-19 Testing’** | |
| **Procedure Code** | **Code Description** |
|  |  |
| 0224U | Antibody, severe acute respiratory syndrome coronavirus 2 (SARS-CoV-2) (Coronavirus disease [COVID-19]), includes titer(s), when performed |
| 0226U | Surrogate viral neutralization test (sVNT), severe acute respiratory syndrome coronavirus 2 (SARS-CoV-2) (Coronavirus disease [COVID-19]), ELISA, plasma, serum |
| 86328 | Immunoassay for infectious agent antibody(ies), qualitative or semiquantitative, single step method (eg, reagent strip); severe acute respiratory syndrome coronavirus 2 (SARS-CoV-2) (Coronavirus disease [COVID-19]) |
| 86408 | Neutralizing antibody, severe acute respiratory syndrome coronavirus 2 (SARS-CoV-2) (Coronavirus disease [COVID-19]); screen |
| 86409 | Neutralizing antibody, severe acute respiratory syndrome coronavirus 2 (SARS-CoV-2) (Coronavirus disease [COVID-19]); titer |
| 86413 | Severe acute respiratory syndrome coronavirus 2 (SARS-CoV-2) (Coronavirus disease [COVID-19]) antibody, quantitative |
| 86769 | Antibody; severe acute respiratory syndrome coronavirus 2 (SARS-CoV-2) (Coronavirus disease [COVID-19]) |
| 87426 | Infectious agent antigen detection by immunoassay technique, (eg, enzyme immunoassay [EIA], enzyme-linked immunosorbent assay [ELISA], immunochemiluminometric assay [IMCA]) qualitative or semiquantitative, multiple-step method; severe acute respiratory sy |
| 0224U | Antibody, severe acute respiratory syndrome coronavirus 2 (SARS-CoV-2) (Coronavirus disease [COVID-19]), includes titer(s), when performed |
| 0226U | Surrogate viral neutralization test (sVNT), severe acute respiratory syndrome coronavirus 2 (SARS-CoV-2) (Coronavirus disease [COVID-19]), ELISA, plasma, serum |
| 86328 | Immunoassay for infectious agent antibody(ies), qualitative or semiquantitative, single step method (eg, reagent strip); severe acute respiratory syndrome coronavirus 2 (SARS-CoV-2) (Coronavirus disease [COVID-19]) |
| 86408 | Neutralizing antibody, severe acute respiratory syndrome coronavirus 2 (SARS-CoV-2) (Coronavirus disease [COVID-19]); screen |
| 86409 | Neutralizing antibody, severe acute respiratory syndrome coronavirus 2 (SARS-CoV-2) (Coronavirus disease [COVID-19]); titer |
| 86413 | Severe acute respiratory syndrome coronavirus 2 (SARS-CoV-2) (Coronavirus disease [COVID-19]) antibody, quantitative |
| 86769 | Antibody; severe acute respiratory syndrome coronavirus 2 (SARS-CoV-2) (Coronavirus disease [COVID-19]) |
| 87426 | Infectious agent antigen detection by immunoassay technique, (eg, enzyme immunoassay [EIA], enzyme-linked immunosorbent assay [ELISA], immunochemiluminometric assay [IMCA]) qualitative or semiquantitative, multiple-step method; severe acute respiratory sy |
| 87635 | Infectious agent detection by nucleic acid (DNA or RNA); severe acute respiratory syndrome coronavirus 2 (SARS-CoV-2) (Coronavirus disease [COVID-19]), amplified probe technique |
| 87636 | Infectious agent detection by nucleic acid (DNA or RNA); severe acute respiratory syndrome coronavirus 2 (SARS-CoV-2) (Coronavirus disease [COVID-19]) and influenza virus types A and B, multiplex amplified probe technique |
| 87637 | Infectious agent detection by nucleic acid (DNA or RNA); severe acute respiratory syndrome coronavirus 2 (SARS-CoV-2) (Coronavirus disease [COVID-19]), influenza virus types A and B, and respiratory syncytial virus, multiplex amplified probe technique |
| 87811 | Infectious agent antigen detection by immunoassay with direct optical (ie, visual) observation; severe acute respiratory syndrome coronavirus 2 (SARS-CoV-2) (Coronavirus disease [COVID-19]) |
| 87913 | Infectious Agent Genotype Analysis By Nucleic Acid (Dna Or Rna); Severe Acute Respiratory Syndrome Coronavirus 2 (Sars-Cov-2) (Coronavirus Disease [Covid-19]), Mutation Identification In Targeted Region(S) |
| C9803 | Hospital outpatient clinic visit specimen collection for Severe Acute Respiratory Syndrome Coronavirus 2 (SARS-CoV-2) (Coronavirus disease [COVID-19]), any specimen source |
| G2023 | Specimen collection for Severe Acute Respiratory Syndrome Coronavirus 2 (SARS-CoV-2) (Coronavirus disease [COVID-19]), any specimen source |
| G2024 | Specimen collection for Severe Acute Respiratory Syndrome Coronavirus 2 (SARS-CoV-2) (Coronavirus disease [COVID-19]) from an individual in a SNF or by a laboratory on behalf of a HHA, any specimen source |
| K1034 | Provision of COVID-19 test, nonprescription self-administered and self-collected use, FDA approved, authorized or cleared, one test count |
| U0002 | 2019-nCoV Coronavirus, SARS-CoV-2/2019-nCoV (COVID-19), any technique, multiple types or subtypes (includes all targets), non-CDC |
| U0003 | Infectious agent detection by nucleic acid (DNA or RNA); Severe Acute Respiratory Syndrome Coronavirus 2 (SARS-CoV-2) (Coronavirus disease [COVID-19]), amplified probe technique, making use of high throughput technologies as described by CMS-2020-01-R |
| U0004 | 2019-nCoV Coronavirus, SARS-CoV-2/2019-nCoV (COVID-19), any technique, multiple types or subtypes (includes all targets), non-CDC, making use of high throughput technologies as described by CMS-2020-01-R |
| U0005 | Infectious agent detection by nucleic acid (DNA or RNA); Severe Acute Respiratory Syndrome Coronavirus 2 (SARS-CoV-2) (Coronavirus disease [COVID-19]), amplified probe technique, CDC or non-CDC, making use of high throughput technologies, completed within |
| **Procedure Codes Used to Define ‘Spirometry’** | |
| **Procedure Code** | **Code Description** |
|  |  |
| 3023F | Spirometry results documented and reviewed (COPD) |
| G8925 | Spirometry test results demonstrate FEV1 >= 60%, FEV1/FVC >= 70%, predicted or patient does not have COPD symptoms |
| G8924 | Spirometry test results demonstrate FEV1/FVC < 70%, FEV < 60% predicted and patient has COPD symptoms (e.g., dyspnea, cough/sputum, wheezing) |
| 3027F | Spirometry test results demonstrate FEV1/FVC greater than or equal to 70% or patient does not have COPD symptoms (COPD) |
| 3025F | Spirometry test results demonstrate FEV1/FVC less than 70% with COPD symptoms (eg, dyspnea, cough/sputum, wheezing) (CAP, COPD) |
| 94010 | Spirometry, including graphic record, total and timed vital capacity, expiratory flow rate measurement(s), with or without maximal voluntary ventilation |
| **Procedure Codes Used to Define ‘Bronchoscopy’** | |
| **Procedure Code** | **Code Description** |
| 00520 | Anesthesia for closed chest procedures; (including bronchoscopy) not otherwise specified |
| 3321 | Bronchoscopy through artificial stoma |
| C7509 | Bronchoscopy, rigid or flexible, diagnostic with cell washing(s) when performed, with computer-assisted image-guided navigation, including fluoroscopic guidance when performed |
| 31622 | Bronchoscopy, rigid or flexible, including fluoroscopic guidance, when performed; diagnostic, with cell washing, when performed (separate procedure) |
| 31637 | Bronchoscopy, rigid or flexible, including fluoroscopic guidance, when performed; each additional major bronchus stented (List separately in addition to code for primary procedure) |
| 31651 | Bronchoscopy, rigid or flexible, including fluoroscopic guidance, when performed; with balloon occlusion, when performed, assessment of air leak, airway sizing, and insertion of bronchial valve(s), each additional lobe (List separately in addition to code for primary procedure[s]) |
| 31647 | Bronchoscopy, rigid or flexible, including fluoroscopic guidance, when performed; with balloon occlusion, when performed, assessment of air leak, airway sizing, and insertion of bronchial valve(s), initial lobe |
| 31634 | Bronchoscopy, rigid or flexible, including fluoroscopic guidance, when performed; with balloon occlusion, with assessment of air leak, with administration of occlusive substance (eg, fibrin glue), if performed |
| 31624 | Bronchoscopy, rigid or flexible, including fluoroscopic guidance, when performed; with bronchial alveolar lavage |
| 31625 | Bronchoscopy, rigid or flexible, including fluoroscopic guidance, when performed; with bronchial or endobronchial biopsy(s), single or multiple sites |
| 0276T | Bronchoscopy, rigid or flexible, including fluoroscopic guidance, when performed; with bronchial thermoplasty, 1 lobe |
| 31660 | Bronchoscopy, rigid or flexible, including fluoroscopic guidance, when performed; with bronchial thermoplasty, 1 lobe |
| 0277T | Bronchoscopy, rigid or flexible, including fluoroscopic guidance, when performed; with bronchial thermoplasty, 2 or more lobes |
| 31661 | Bronchoscopy, rigid or flexible, including fluoroscopic guidance, when performed; with bronchial thermoplasty, 2 or more lobes |
| 31623 | Bronchoscopy, rigid or flexible, including fluoroscopic guidance, when performed; with brushing or protected brushings |
| 31627 | Bronchoscopy, rigid or flexible, including fluoroscopic guidance, when performed; with computer-assisted, image-guided navigation (List separately in addition to code for primary procedure[s]) |
| 31641 | Bronchoscopy, rigid or flexible, including fluoroscopic guidance, when performed; with destruction of tumor or relief of stenosis by any method other than excision (eg, laser therapy, cryotherapy) |
| 31653 | Bronchoscopy, rigid or flexible, including fluoroscopic guidance, when performed; with endobronchial ultrasound (EBUS) guided transtracheal and/or transbronchial sampling (eg, aspiration[s]/biopsy[ies]), 3 or more mediastinal and/or hilar lymph node stations or structures |
| 31652 | Bronchoscopy, rigid or flexible, including fluoroscopic guidance, when performed; with endobronchial ultrasound (EBUS) guided transtracheal and/or transbronchial sampling (eg, aspiration[s]/biopsy[ies]), one or two mediastinal and/or hilar lymph node stations or structures |
| 31640 | Bronchoscopy, rigid or flexible, including fluoroscopic guidance, when performed; with excision of tumor |
| 31656 | Bronchoscopy, rigid or flexible, including fluoroscopic guidance, when performed; with injection of contrast material for segmental bronchography (fiberscope only) |
| 31636 | Bronchoscopy, rigid or flexible, including fluoroscopic guidance, when performed; with placement of bronchial stent(s) (includes tracheal/bronchial dilation as required), initial bronchus |
| 31643 | Bronchoscopy, rigid or flexible, including fluoroscopic guidance, when performed; with placement of catheter(s) for intracavitary radioelement application |
| 31626 | Bronchoscopy, rigid or flexible, including fluoroscopic guidance, when performed; with placement of fiducial markers, single or multiple |
| 31631 | Bronchoscopy, rigid or flexible, including fluoroscopic guidance, when performed; with placement of tracheal stent(s) (includes tracheal/bronchial dilation as required) |
| 0252T | Bronchoscopy, rigid or flexible, including fluoroscopic guidance, when performed; with removal of bronchial valve(s), each additional lobe (List separately in addition to code for primary procedure) |
| 31649 | Bronchoscopy, rigid or flexible, including fluoroscopic guidance, when performed; with removal of bronchial valve(s), each additional lobe (List separately in addition to code for primary procedure) |
| 0251T | Bronchoscopy, rigid or flexible, including fluoroscopic guidance, when performed; with removal of bronchial valve(s), initial lobe |
| 31648 | Bronchoscopy, rigid or flexible, including fluoroscopic guidance, when performed; with removal of bronchial valve(s), initial lobe |
| 31635 | Bronchoscopy, rigid or flexible, including fluoroscopic guidance, when performed; with removal of foreign body |
| 31638 | Bronchoscopy, rigid or flexible, including fluoroscopic guidance, when performed; with revision of tracheal or bronchial stent inserted at previous session (includes tracheal/bronchial dilation as required) |
| 31645 | Bronchoscopy, rigid or flexible, including fluoroscopic guidance, when performed; with therapeutic aspiration of tracheobronchial tree, initial |
| 31646 | Bronchoscopy, rigid or flexible, including fluoroscopic guidance, when performed; with therapeutic aspiration of tracheobronchial tree, subsequent, same hospital stay |
| 31630 | Bronchoscopy, rigid or flexible, including fluoroscopic guidance, when performed; with tracheal/bronchial dilation or closed reduction of fracture |
| 31632 | Bronchoscopy, rigid or flexible, including fluoroscopic guidance, when performed; with transbronchial lung biopsy(s), each additional lobe (List separately in addition to code for primary procedure) |
| 31628 | Bronchoscopy, rigid or flexible, including fluoroscopic guidance, when performed; with transbronchial lung biopsy(s), single lobe |
| 31633 | Bronchoscopy, rigid or flexible, including fluoroscopic guidance, when performed; with transbronchial needle aspiration biopsy(s), each additional lobe (List separately in addition to code for primary procedure) |
| 31629 | Bronchoscopy, rigid or flexible, including fluoroscopic guidance, when performed; with transbronchial needle aspiration biopsy(s), trachea, main stem and/or lobar bronchus(i) |
| 31654 | Bronchoscopy, rigid or flexible, including fluoroscopic guidance, when performed; with transendoscopic endobronchial ultrasound (EBUS) during bronchoscopic diagnostic or therapeutic intervention(s) for peripheral lesion(s) (List separately in addition to code for primary procedure[s]) |
| C9751 | Bronchoscopy, rigid or flexible, transbronchial ablation of lesion(s) by microwave energy, including fluoroscopic guidance, when performed, with computed tomography acquisition(s) and 3D rendering, computer-assisted, image-guided navigation, and endobronchial ultrasound (EBUS) guided transtracheal and/or transbronchial sampling (e.g., aspiration[s]/biopsy[ies]) and all mediastinal and/or hilar lymph node stations or structures and therapeutic intervention(s) |
| C7510 | Bronchoscopy, rigid or flexible, with bronchial alveolar lavage(s), with computer-assisted image-guided navigation, including fluoroscopic guidance when performed |
| 0781T | Bronchoscopy, rigid or flexible, with insertion of esophageal protection device and circumferential radiofrequency destruction of the pulmonary nerves, including fluoroscopic guidance when performed; bilateral mainstem bronchi |
| 0782T | Bronchoscopy, rigid or flexible, with insertion of esophageal protection device and circumferential radiofrequency destruction of the pulmonary nerves, including fluoroscopic guidance when performed; unilateral mainstem bronchus |
| C7511 | Bronchoscopy, rigid or flexible, with single or multiple bronchial or endobronchial biopsy(ies), single or multiple sites, with computer-assisted image-guided navigation, including fluoroscopic guidance when performed |
| C7512 | Bronchoscopy, rigid or flexible, with single or multiple bronchial or endobronchial biopsy(ies), single or multiple sites, with transendoscopic endobronchial ultrasound (EBUS) during bronchoscopic diagnostic or therapeutic intervention(s) for peripheral lesion(s), including fluoroscopic guidance when performed |
| 3322 | Fiber-optic bronchoscopy |
| 3323 | Other bronchoscopy |
| 31615 | Tracheobronchoscopy through established tracheostomy incision |
| **Procedure Codes Used to Define ‘Mobility Assistance’** | |
| **Procedure Code** | **Code Description** |
| 29355 | Application of long leg cast (thigh to toes); walker or ambulatory type |
| 97542 | Wheelchair management (eg, assessment, fitting, training), each 15 minutes |
| A0130 | Nonemergency transportation: wheelchair van |
| A4637 | Replacement, tip, cane, crutch, walker, each |
| E0100 | Cane, includes canes of all materials, adjustable or fixed, with tip |
| E0105 | Cane, quad or three-prong, includes canes of all materials, adjustable or fixed, with tips |
| E0110 | Crutches, forearm, includes crutches of various materials, adjustable or fixed, pair, complete with tips and handgrips |
| E0114 | Crutches, underarm, other than wood, adjustable or fixed, pair, with pads, tips, and handgrips |
| E0118 | Crutch substitute, lower leg platform, with or without wheels, each |
| E0135 | Walker, folding (pickup), adjustable or fixed height |
| E0141 | Walker, rigid, wheeled, adjustable or fixed height |
| E0143 | Walker, folding, wheeled, adjustable or fixed height |
| E0144 | Walker, enclosed, four-sided framed, rigid or folding, wheeled with posterior seat |
| E0147 | Walker, heavy-duty, multiple braking system, variable wheel resistance |
| E0148 | Walker, heavy-duty, without wheels, rigid or folding, any type, each |
| E0149 | Walker, heavy-duty, wheeled, rigid or folding, any type |
| E0154 | Platform attachment, walker, each |
| E0155 | Wheel attachment, rigid pick-up walker, per pair |
| E0156 | Seat attachment, walker |
| E0159 | Brake attachment for wheeled walker, replacement, each |
| E0163 | Commode chair, mobile or stationary, with fixed arms |
| E0165 | Commode chair, mobile or stationary, with detachable arms |
| E0168 | Commode chair, extra wide and/or heavy-duty, stationary or mobile, with or without arms, any type, each |
| E0950 | Wheelchair accessory, tray, each |
| E0954 | Wheelchair accessory, foot box, any type, includes attachment and mounting hardware, each foot |
| E0955 | Wheelchair accessory, headrest, cushioned, any type, including fixed mounting hardware, each |
| E0956 | Wheelchair accessory, lateral trunk or hip support, any type, including fixed mounting hardware, each |
| E0960 | Wheelchair accessory, shoulder harness/straps or chest strap, including any type mounting hardware |
| E0961 | Manual wheelchair accessory, wheel lock brake extension (handle), each |
| E0971 | Manual wheelchair accessory, antitipping device, each |
| E0973 | Wheelchair accessory, adjustable height, detachable armrest, complete assembly, each |
| E0978 | Wheelchair accessory, positioning belt/safety belt/pelvic strap, each |
| E0981 | Wheelchair accessory, seat upholstery, replacement only, each |
| E0982 | Wheelchair accessory, back upholstery, replacement only, each |
| E0986 | Manual wheelchair accessory, push-rim activated power assist system |
| E0990 | Wheelchair accessory, elevating legrest, complete assembly, each |
| E0992 | Manual wheelchair accessory, solid seat insert |
| E0995 | Wheelchair accessory, calf rest/pad, replacement only, each |
| E1002 | Wheelchair accessory, power seating system, tilt only |
| E1005 | Wheelchair accessory, power seating system, recline only, with power shear reduction |
| E1007 | Wheelchair accessory, power seating system, combination tilt and recline, with mechanical shear reduction |
| E1008 | Wheelchair accessory, power seating system, combination tilt and recline, with power shear reduction |
| E1012 | Wheelchair accessory, addition to power seating system, center mount power elevating leg rest/platform, complete system, any type, each |
| E1014 | Reclining back, addition to pediatric size wheelchair |
| E1020 | Residual limb support system for wheelchair, any type |
| E1028 | Wheelchair accessory, manual swingaway, retractable or removable mounting hardware for joystick, other control interface or positioning accessory |
| E1029 | Wheelchair accessory, ventilator tray, fixed |
| E1140 | Wheelchair, detachable arms, desk or full-length, swing-away detachable footrests |
| E1161 | Manual adult size wheelchair, includes tilt in space |
| E1225 | Wheelchair accessory, manual semi-reclining back, (recline greater than 15 degrees, but less than 80 degrees), each |
| E1226 | Wheelchair accessory, manual fully reclining back, (recline greater than 80 degrees), each |
| E1232 | Wheelchair, pediatric size, tilt-in-space, folding, adjustable, with seating system |
| E1234 | Wheelchair, pediatric size, tilt-in-space, folding, adjustable, without seating system |
| E1236 | Wheelchair, pediatric size, folding, adjustable, with seating system |
| E2201 | Manual wheelchair accessory, nonstandard seat frame, width greater than or equal to 20 in and less than 24 in |
| E2206 | Manual wheelchair accessory, wheel lock assembly, complete, replacement only, each |
| E2208 | Wheelchair accessory, cylinder tank carrier, each |
| E2210 | Wheelchair accessory, bearings, any type, replacement only, each |
| E2211 | Manual wheelchair accessory, pneumatic propulsion tire, any size, each |
| E2213 | Manual wheelchair accessory, insert for pneumatic propulsion tire (removable), any type, any size, each |
| E2214 | Manual wheelchair accessory, pneumatic caster tire, any size, each |
| E2218 | Manual wheelchair accessory, foam propulsion tire, any size, each |
| E2226 | Manual wheelchair accessory, caster fork, any size, replacement only, each |
| E2228 | Manual wheelchair accessory, wheel braking system and lock, complete, each |
| E2231 | Manual wheelchair accessory, solid seat support base (replaces sling seat), includes any type mounting hardware |
| E2292 | Seat, planar, for pediatric size wheelchair including fixed attaching hardware |
| E2293 | Back, contoured, for pediatric size wheelchair including fixed attaching hardware |
| E2300 | Wheelchair accessory, power seat elevation system, any type |
| E2310 | Power wheelchair accessory, electronic connection between wheelchair controller and one power seating system motor, including all related electronics, indicator feature, mechanical function selection switch, and fixed mounting hardware |
| E2311 | Power wheelchair accessory, electronic connection between wheelchair controller and 2 or more power seating system motors, including all related electronics, indicator feature, mechanical function selection switch, and fixed mounting hardware |
| E2313 | Power wheelchair accessory, harness for upgrade to expandable controller, including all fasteners, connectors and mounting hardware, each |
| E2323 | Power wheelchair accessory, specialty joystick handle for hand control interface, prefabricated |
| E2359 | Power wheelchair accessory, group 34 sealed lead acid battery, each (e.g., gel cell, absorbed glass mat) |
| E2361 | Power wheelchair accessory, 22 NF sealed lead acid battery, each (e.g., gel cell, absorbed glassmat) |
| E2363 | Power wheelchair accessory, group 24 sealed lead acid battery, each (e.g., gel cell, absorbed glassmat) |
| E2365 | Power wheelchair accessory, U-1 sealed lead acid battery, each (e.g., gel cell, absorbed glassmat) |
| E2366 | Power wheelchair accessory, battery charger, single mode, for use with only one battery type, sealed or nonsealed, each |
| E2370 | Power wheelchair component, integrated drive wheel motor and gear box combination, replacement only |
| E2374 | Power wheelchair accessory, hand or chin control interface, standard remote joystick (not including controller), proportional, including all related electronics and fixed mounting hardware, replacement only |
| E2375 | Power wheelchair accessory, nonexpandable controller, including all related electronics and mounting hardware, replacement only |
| E2376 | Power wheelchair accessory, expandable controller, including all related electronics and mounting hardware, replacement only |
| E2377 | Power wheelchair accessory, expandable controller, including all related electronics and mounting hardware, upgrade provided at initial issue |
| E2381 | Power wheelchair accessory, pneumatic drive wheel tire, any size, replacement only, each |
| E2382 | Power wheelchair accessory, tube for pneumatic drive wheel tire, any size, replacement only, each |
| E2386 | Power wheelchair accessory, foam filled drive wheel tire, any size, replacement only, each |
| E2390 | Power wheelchair accessory, solid (rubber/plastic) drive wheel tire, any size, replacement only, each |
| E2391 | Power wheelchair accessory, solid (rubber/plastic) caster tire (removable), any size, replacement only, each |
| E2392 | Power wheelchair accessory, solid (rubber/plastic) caster tire with integrated wheel, any size, replacement only, each |
| E2394 | Power wheelchair accessory, drive wheel excludes tire, any size, replacement only, each |
| E2601 | General use wheelchair seat cushion, width less than 22 in, any depth |
| E2602 | General use wheelchair seat cushion, width 22 in or greater, any depth |
| E2603 | Skin protection wheelchair seat cushion, width less than 22 in, any depth |
| E2605 | Positioning wheelchair seat cushion, width less than 22 in, any depth |
| E2606 | Positioning wheelchair seat cushion, width 22 in or greater, any depth |
| E2607 | Skin protection and positioning wheelchair seat cushion, width less than 22 in, any depth |
| E2608 | Skin protection and positioning wheelchair seat cushion, width 22 in or greater, any depth |
| E2609 | Custom fabricated wheelchair seat cushion, any size |
| E2611 | General use wheelchair back cushion, width less than 22 in, any height, including any type mounting hardware |
| E2612 | General use wheelchair back cushion, width 22 in or greater, any height, including any type mounting hardware |
| E2613 | Positioning wheelchair back cushion, posterior, width less than 22 in, any height, including any type mounting hardware |
| E2615 | Positioning wheelchair back cushion, posterior-lateral, width less than 22 in, any height, including any type mounting hardware |
| E2617 | Custom fabricated wheelchair back cushion, any size, including any type mounting hardware |
| E2619 | Replacement cover for wheelchair seat cushion or back cushion, each |
| E2620 | Positioning wheelchair back cushion, planar back with lateral supports, width less than 22 in, any height, including any type mounting hardware |
| E2621 | Positioning wheelchair back cushion, planar back with lateral supports, width 22 in or greater, any height, including any type mounting hardware |
| E2622 | Skin protection wheelchair seat cushion, adjustable, width less than 22 in, any depth |
| E2623 | Skin protection wheelchair seat cushion, adjustable, width 22 in or greater, any depth |
| E2624 | Skin protection and positioning wheelchair seat cushion, adjustable, width less than 22 in, any depth |
| E2625 | Skin protection and positioning wheelchair seat cushion, adjustable, width 22 in or greater, any depth |
| G8978 | Mobility: walking and moving around functional limitation, current status, at therapy episode outset and at reporting intervals |
| G8979 | Mobility: walking and moving around functional limitation, projected goal status, at therapy episode outset, at reporting intervals, and at discharge or to end reporting |
| G8980 | Mobility: walking and moving around functional limitation, discharge status, at discharge from therapy or to end reporting |
| K0001 | Standard wheelchair |
| K0002 | Standard hemi (low seat) wheelchair |
| K0003 | Lightweight wheelchair |
| K0004 | High strength, lightweight wheelchair |
| K0005 | Ultralightweight wheelchair |
| K0006 | Heavy-duty wheelchair |
| K0007 | Extra heavy-duty wheelchair |
| K0011 | Standard-weight frame motorized/power wheelchair with programmable control parameters for speed adjustment, tremor dampening, acceleration control and braking |
| K0108 | Wheelchair component or accessory, not otherwise specified |
| K0195 | Elevating legrests, pair (for use with capped rental wheelchair base) |
| K0733 | Power wheelchair accessory, 12 to 24 amp hour sealed lead acid battery, each (e.g., gel cell, absorbed glassmat) |
| K0821 | Power wheelchair, group 2 standard, portable, captain's chair, patient weight capacity up to and including 300 pounds |
| K0822 | Power wheelchair, group 2 standard, sling/solid seat/back, patient weight capacity up to and including 300 pounds |
| K0823 | Power wheelchair, group 2 standard, captain's chair, patient weight capacity up to and including 300 pounds |
| K0843 | Power wheelchair, group 2 heavy-duty, multiple power option, sling/solid seat/back, patient weight capacity 301 to 450 pounds |
| K0856 | Power wheelchair, group 3 standard, single power option, sling/solid seat/back, patient weight capacity up to and including 300 pounds |
| K0858 | Power wheelchair, group 3 heavy-duty, single power option, sling/solid seat/back, patient weight 301 to 450 pounds |
| K0861 | Power wheelchair, group 3 standard, multiple power option, sling/solid seat/back, patient weight capacity up to and including 300 pounds |
| K0862 | Power wheelchair, group 3 heavy-duty, multiple power option, sling/solid seat/back, patient weight capacity 301 to 450 pounds |
| **Procedure Codes Used to Define ‘Bronchial Alveolar Lavage (BAL)’** | |
| **Procedure Code** | **Code Description** |
| 31624 | Bronchoscopy, rigid or flexible, including fluoroscopic guidance, when performed; with bronchial alveolar lavage |
| C7510 | Bronchoscopy, rigid or flexible, with bronchial alveolar lavage(s), with computer-assisted image-guided navigation, including fluoroscopic guidance when performed |
| **Procedure Codes Used to Define ‘Lung Lavage’** | |
| **Procedure Code** | **Code Description** |
| 32001 | Total lung lavage (unilateral) |
| 32997 | Total lung lavage (unilateral) |
| **Procedure Codes Used to Define ‘Thoracoscopy”** | |
| **Procedure Code** | **Code Description** |
|  |  |
| 00528 | Anesthesia for closed chest procedures; mediastinoscopy and diagnostic thoracoscopy not utilizing 1 lung ventilation |
| 00529 | Anesthesia for closed chest procedures; mediastinoscopy and diagnostic thoracoscopy utilizing 1 lung ventilation |
| 00540 | Anesthesia for thoracotomy procedures involving lungs, pleura, diaphragm, and mediastinum (including surgical thoracoscopy); not otherwise specified |
| 00541 | Anesthesia for thoracotomy procedures involving lungs, pleura, diaphragm, and mediastinum (including surgical thoracoscopy); utilizing 1 lung ventilation |
| 00542 | Anesthesia for thoracotomy procedures involving lungs, pleura, diaphragm, and mediastinum (including surgical thoracoscopy); decortication |
| 00544 | Anesthesia for thoracotomy procedures involving lungs, pleura, diaphragm, and mediastinum (including surgical thoracoscopy); pleurectomy |
| 00546 | Anesthesia for thoracotomy procedures involving lungs, pleura, diaphragm, and mediastinum (including surgical thoracoscopy); pulmonary resection with thoracoplasty |
| 00548 | Anesthesia for thoracotomy procedures involving lungs, pleura, diaphragm, and mediastinum (including surgical thoracoscopy); intrathoracic procedures on the trachea and bronchi |
| 32601 | Thoracoscopy, diagnostic (separate procedure); lungs, pericardial sac, mediastinal or pleural space, without biopsy |
| 32602 | Thoracoscopy, diagnostic (separate procedure); lungs and pleural space, with biopsy |
| 32603 | Thoracoscopy, diagnostic (separate procedure); pericardial sac, without biopsy |
| 32604 | Thoracoscopy, diagnostic (separate procedure); pericardial sac, with biopsy |
| 32605 | Thoracoscopy, diagnostic (separate procedure); mediastinal space, without biopsy |
| 32606 | Thoracoscopy, diagnostic (separate procedure); mediastinal space, with biopsy |
| 32607 | Thoracoscopy; with diagnostic biopsy(ies) of lung infiltrate(s) (eg, wedge, incisional), unilateral |
| 32608 | Thoracoscopy; with diagnostic biopsy(ies) of lung nodule(s) or mass(es) (eg, wedge, incisional), unilateral |
| 32609 | Thoracoscopy; with biopsy(ies) of pleura |
| 32650 | Thoracoscopy, surgical; with pleurodesis (eg, mechanical or chemical) |
| 32651 | Thoracoscopy, surgical; with partial pulmonary decortication |
| 32652 | Thoracoscopy, surgical; with total pulmonary decortication, including intrapleural pneumonolysis |
| 32653 | Thoracoscopy, surgical; with removal of intrapleural foreign body or fibrin deposit |
| 32654 | Thoracoscopy, surgical; with control of traumatic hemorrhage |
| 32655 | Thoracoscopy, surgical; with resection-plication of bullae, includes any pleural procedure when performed |
| 32656 | Thoracoscopy, surgical; with parietal pleurectomy |
| 32657 | Thoracoscopy, surgical; with wedge resection of lung, single or multiple |
| 32658 | Thoracoscopy, surgical; with removal of clot or foreign body from pericardial sac |
| 32659 | Thoracoscopy, surgical; with creation of pericardial window or partial resection of pericardial sac for drainage |
| 32660 | Thoracoscopy, surgical; with total pericardiectomy |
| 32661 | Thoracoscopy, surgical; with excision of pericardial cyst, tumor, or mass |
| 32662 | Thoracoscopy, surgical; with excision of mediastinal cyst, tumor, or mass |
| 32663 | Thoracoscopy, surgical; with lobectomy (single lobe) |
| 32664 | Thoracoscopy, surgical; with thoracic sympathectomy |
| 32665 | Thoracoscopy, surgical; with esophagomyotomy (Heller type) |
| 32666 | Thoracoscopy, surgical; with therapeutic wedge resection (eg, mass, nodule), initial unilateral |
| 32667 | Thoracoscopy, surgical; with therapeutic wedge resection (eg, mass or nodule), each additional resection, ipsilateral (List separately in addition to code for primary procedure) |
| 32668 | Thoracoscopy, surgical; with diagnostic wedge resection followed by anatomic lung resection (List separately in addition to code for primary procedure) |
| 32669 | Thoracoscopy, surgical; with removal of a single lung segment (segmentectomy) |
| 32670 | Thoracoscopy, surgical; with removal of two lobes (bilobectomy) |
| 32671 | Thoracoscopy, surgical; with removal of lung (pneumonectomy) |
| 32672 | Thoracoscopy, surgical; with resection-plication for emphysematous lung (bullous or non-bullous) for lung volume reduction (LVRS), unilateral includes any pleural procedure, when performed |
| 32673 | Thoracoscopy, surgical; with resection of thymus, unilateral or bilateral |
| 32674 | Thoracoscopy, surgical; with mediastinal and regional lymphadenectomy (List separately in addition to code for primary procedure) |
